# Brachytherapy Workflow Practices: Analysis of Different Workflow Scenarios in Patients With Cervical Cancer and Impact on IGBT Implementation—An IAEA Study

**DOI:** 10.1200/GO.23.00336

**Published:** 2024-02-22

**Authors:** Sandra Ndarukwa, Jerickson Abbie Flores, Eduardo Rosenblatt, Daniel Berger, Kamal Akbarov, Natasha Hedden, Supriya Chopra, Varsha Hande, Alfredo Polo Rubio

**Affiliations:** ^1^ Applied Radiobiology and Radiotherapy Section, Division of Human Health, International Atomic Energy Agency, Vienna, Austria; ^2^ Section of Dosimetry and Medical Radiation Physics, Division of Human Health, International Atomic Energy Agency, Vienna, Austria; ^3^ Advanced Centre for Treatment Research and Education in Cancer (ACTREC), Tata Memorial Centre, Homi Bhabha National Institute, Mumbai, India; ^4^ City Cancer Challenge, Technical Cooperation and Capacity Development, Geneva, Switzerland

## Abstract

**PURPOSE:**

The workflow of brachytherapy (BT) is an essential aspect of treatment to consider in image-guided brachytherapy (IGBT). It has an overarching effect influencing patient throughput and the number of cancer treatments that can be performed as it occupies equipment, space, and personnel. There is limited research addressing this issue. Under the International Atomic Energy Agency's Coordinated Research Activity titled IGBT for cervix cancer: An implementation study, our study analyzes various scenarios in the clinical workflow of BT delivery for cervical cancer. It aims to determine the extent to which these scenarios allow the routine implementation of IGBT. With this information, current barriers and individualized adaptations to efficient workflows can be identified to enhance the global application of IGBT, leading to better cervical cancer treatment.

**MATERIALS AND METHODS:**

A web-based poll of questions regarding practices in BT workflow was presented to 62 participants from low-, lower middle-, upper middle-, and high-income countries (19 countries).

**RESULTS:**

This study highlighted diversity in BT practices across countries, income levels, and regions. It identified variations in workflow, patient throughput, and resource availability, which can have implications for the efficiency and quality of BT treatments. Scenario A, utilizing multiple locations for the steps of the BT procedure, was the most commonly used. The availability of resources, such as imaging devices and trained personnel, varied among the participating centers and remained challenging for IGBT implementation and sustainability.

**CONCLUSION:**

The design of the BT facility plays a vital role in improving efficiency, with a dedicated BT suite contributing to an efficient workflow but limiting patient throughput, especially for high-volume centers. Although IGBT is effective, its implementation requires consideration of various logistical challenges and should be individualized.

## INTRODUCTION

Cervical cancer is the fourth most common cancer among women globally, and its incidence has increased by 33% over the past 10 years.^1,2^ In 2020, 341,831 cancer deaths were attributed to women with cervical uteri cancer, according to GLOBOCAN data.^1^ Approximately 90% of these deaths occurred in low- and middle-income countries (LMICs), mainly in sub-Saharan Africa, where most women present with advanced disease.^1^ Cervical cancer is a significant public health problem, and global efforts toward the reduction of cervical cancer incidence and mortality are of paramount importance. In 2020, the WHO launched the Cervical Cancer Elimination Initiative, which led to the global strategy to eliminate cervical cancer as a public health problem with a threshold of four cases per 100,000 women within the 21st century.^3^ The elimination initiative targets that by 2030, 90% of girls age younger than 15 years will be fully vaccinated, 70% of women age 35-45 years will be screened with high-performance tests, and 90% of women with cervical cancer will receive appropriate treatment (90-70-90 targets). This idea relies on three main pillars: prevent, screen, and treat, capturing a comprehensive approach that includes prevention, effective screening and treatment of precancerous lesions, early cancer diagnosis, and programs for managing invasive cancer.^3^ In countries with high cervical cancer incidence, tremendous effort will be needed to overcome the challenges.

**CONTEXT**

**Key Objective**
Do brachytherapy (BT) workflow practices affect implementation of image-guided brachytherapy (IGBT)? IGBT is associated with improved local control and reduced treatment-related toxicity. Workflow practices including the design of the operating rooms play an important role in the implementation of IGBT. Utilization of a dedicated BT suite is considered most efficient; however, performing of various steps of the BT workflow in multiple rooms offers the advantage of parallel patient preparation, thereby increasing patient throughput.
**Knowledge Generated**
The use of multiple rooms allows overlapping of treatment steps resulting in higher patient throughput. Availability of resources, such as imaging devices and trained personnel, is important for IGBT implementation.
**Relevance**
Our findings add to the evidence regarding impact of workflow practices on IGBT implementation. The use of multiple rooms could be considered for high volume centers in low- and middle-income countries to improve efficiency thus allowing IGBT implementation. Careful consideration of individual institutions' specific needs and limitations is important to ensure safe and efficient implementation of IGBT.

Radiotherapy is an essential component in the treatment of cervical cancer, particularly for locally advanced cervical cancer.^4^ As part of multimodality treatment, brachytherapy (BT) has an important and established role in the curative management of cervical cancer in combination with external beam radiation therapy and concurrent chemotherapy. Several studies have shown that combined intracavitary BT and chemoradiation in cervical cancer leads to lower in-field failure rates and improved cancer-specific and overall survival rates compared with treatments without BT.^5^ The use of three-dimensional (3D) BT techniques has shown a trend toward increased local control and improved overall survival with reduced toxicity compared with the conventional two-dimensional (2D) BT technique.^6,7^ Recent meta-analyses have shown that image-guided brachytherapy (IGBT) has potential benefits on important clinical outcomes such as local recurrence-free survival and progression-free survival compared with conventional point A-based BT.^8,9^ The multicenter study, EMBRACE I, evaluating 3D-BT in locally advanced cervical cancer reported a local failure rate of 6.5% (crude rate) with a median follow-up of 25 months and grade 3 or 4 late rectal morbidity in <2% of patients.^10,11^ Another prospective trial from France, comparing 2D-BT and 3D-BT, concluded that 3D-BT improved local control with half the toxicity observed with 2D-BT.^12^ Therefore, 3D-BT should be considered in the standard curative management of cervical cancer.^4^ Recommendations for computed tomography (CT)–based contouring in image-guided adaptive BT for cervical cancer were developed by experts.^13^

The implementation of IGBT is a multistep process that necessitates significant upscaling of the departmental workflow.^14^ This upscaling includes incorporating concepts that clinicians and physicists may be unfamiliar with and implementing logistical and practical requirements necessary to ensure safe and secure radiotherapy delivery, such as quality assurance programs and image guidance systems.^15^ There are several factors limiting implementation, such as a lack of access to CT/magnetic resonance imaging (MRI) machines, CT/MRI-compatible applicators, knowledge of new planning software, operating theaters, BT physicists, and insufficient experience of CT/MRI interpretation for tumor delineation.^16^ Another aspect affecting implementation is the organizational setup of the department, including the building layout and other physical factors, such as limited space, which might affect the routine use of IGBT. Patients might need to be moved to multiple locations, such as the radiology department for imaging and a separate treatment room for planning and treatment delivery after the applicator is placed. At the same time, other designs keep the BT patient in one room for all purposes. This affects the patient's workflow and the overall feasibility and efficiency of the IGBT process. To streamline the patient's journey from admission through theater, imaging, planning, treatment delivery, and follow-up, it is essential to have an optimized workflow. This can be achieved by carefully considering the number of rooms and the layout within the BT department, which can significantly enhance the multidisciplinary approach to effective patient care.

The workflow of BT for cervical cancer treatment can be divided into nine essential steps: (1) clinical assessment of the patient, (2) appropriate applicator and implantation technique selection, (3) applicator insertion, (4) imaging of the patient with applicators in situ for treatment planning, (5) treatment planning, (6) dose delivery, (7) applicator removal, (8) clinical evaluation to assess the patient, and (9) patient discharge. These steps are consistent across all levels of BT (from level 2-3: radiograph-based 2D, CT-based, or even MRI–based “3D”), with variations in the skill sets and clinical responsibility of personnel.^17^

In this study, four scenarios covering the essential workflow steps of BT of the cervix are evaluated to assess clinical practices in different global regions noting variations in where these essential steps occur within the cancer centers. Essential information can be obtained regarding various practices to identify room for optimization of IGBT implementation strategies on the basis of different settings and infrastructural limitations, thereby enhancing patient throughput efficiency.

## MATERIALS AND METHODS

An online questionnaire and a focus discussion on workflow scenarios and clinical practice in cervical cancer BT were conducted among 62 participants from various regions and income levels during a BT Virtual Tumor Board.

**FIG 1:**
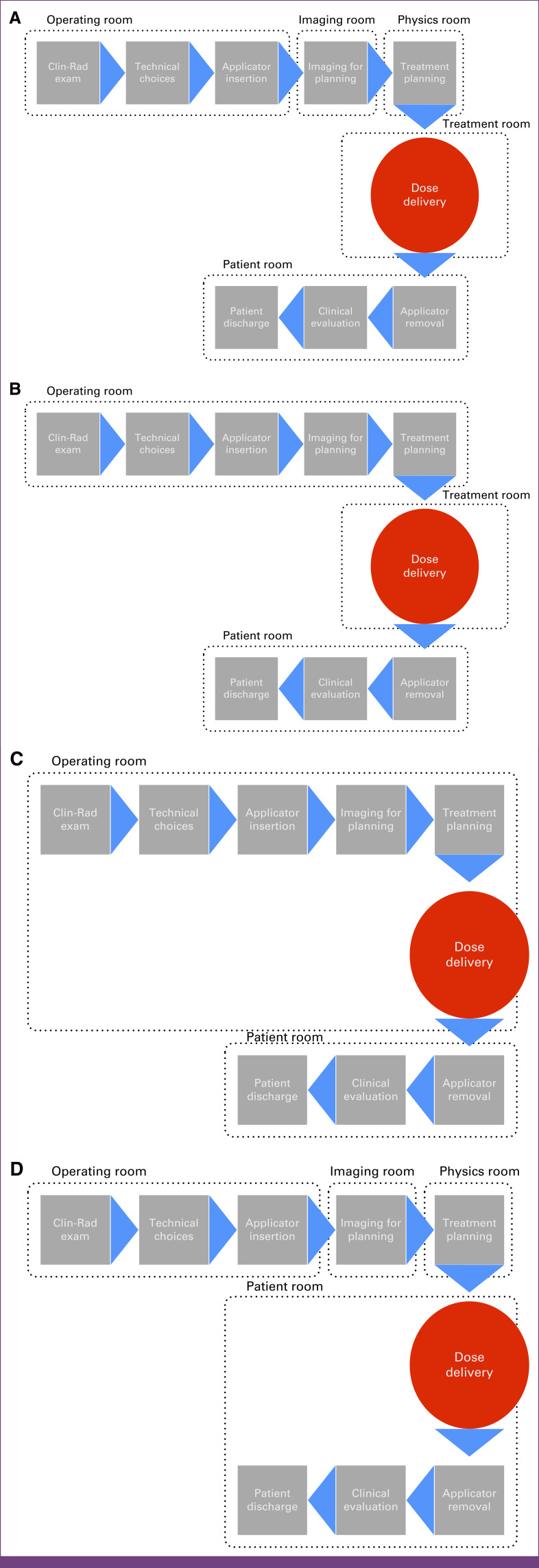
Illustration of four BT workflow scenarios based on infrastructure with the use of (A) operating, imaging, physics, treatment, and patient rooms; (B) operating, treatment, and patient rooms; (C) operating and patient room; and (D) operating, imaging, physics, and patient rooms. BT, brachytherapy; Clin-Rad exam, clinical and radiologic examination.

The questionnaire was used to obtain information regarding various workflow scenarios by asking participants to describe the sequences of steps leading to the delivery of BT in their departments, including details about infrastructure layout, work schedules, treatment delivery practices, and availability of anesthesiologists (Fig 1).

Scenario A described a workflow where clinical examination, discussion about the technical choice of treatment, and applicator insertion were performed in the operating room; imaging for planning was done in the imaging room; and treatment planning was performed in the physics room. The dose was delivered in a treatment room, and applicator removal, clinical evaluation, and discharge were done in a patient recovery room. Scenario B described a process where all actions up to treatment planning were performed in the operating room, the BT dose was delivered in the treatment room, and applicator removal and discharge were conducted in the patient room. For scenario C (BT suite), all procedures up to dose delivery were performed in the operating room, followed by applicator removal and patient discharge, which occurred in the patient room. Scenario D describes a setup similar to A up to treatment planning, following which dose delivery, applicator removal, and patient discharge were performed in the patient room.

BT experts facilitated deeper discussions on these workflow scenarios during the Virtual Tumor Board (Table 1).

**TABLE 1:** The Questions Posed to Participants Are Listed in Order of Appearance

No.	Questionnaire	Answers
1	How many BT patients do you treat per day?	Two or less Three to four Five to six More than six
2	Which workflow best describes your current practice?	Scenario A Scenario B Scenario C Scenario D
3	Does your BT workflow include overnight inpatient stays?	Yes No
4	At what time do you start your first BT procedure?	6-8 am 8-10 am 10-12 pm 12-2 pm 2-4 pm
5	When do you start the BT procedure for your next patient?	Parallel with previous patient applicator insertion After the previous patient applicator insertion After previous patient treatment planning After previous patient dose delivery After the previous patient applicator removal
6	How many hours does your BT unit operate? (from workflow start of first patient to workflow end of last patient)	Less than 4 hours 4-8 hours 8-12 hours ≥12 hours
7	Do you have an anesthesiologist assigned to BT procedures?	Yes (all patients) Yes (intracavitary patients only) Yes (interstitial patients only) Yes (in exceptional cases only)

Abbreviation: BT, brachytherapy.

## RESULTS

**FIG 2:**
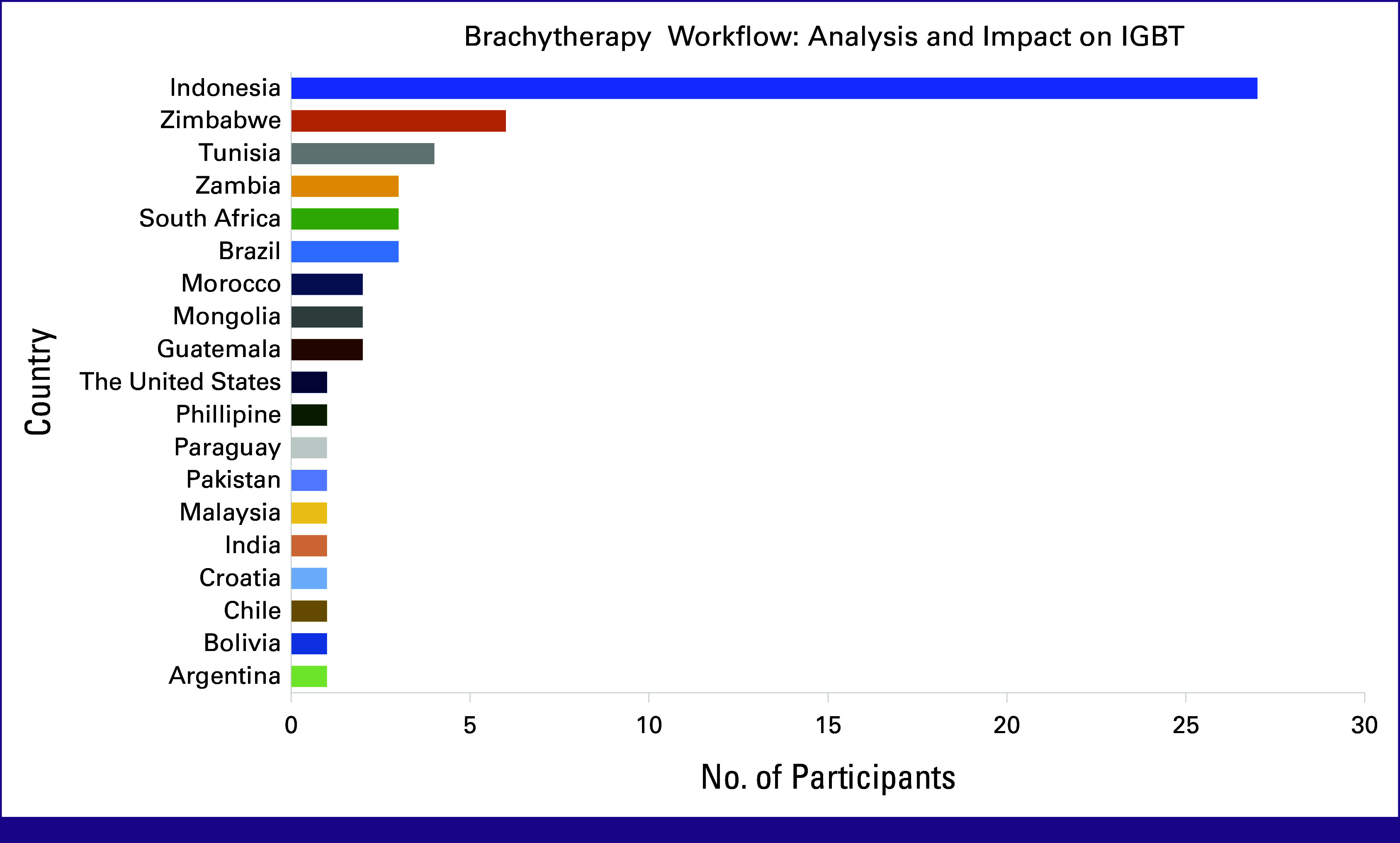
The origin countries of participants were recorded to determine geographical representation, with the most prevalent country highlighted. IGBT, image-guided brachytherapy.

Nineteen countries were represented, with 43% of the participants from Indonesia, as shown in Figure 2. Zimbabwe accounted for approximately 10% of the overall participants, followed by Tunisia (6% of participants). Each of the remaining 16 countries constituted <5% of all participants.

Most participants were from LMICs, as shown in Table 2.

**TABLE 2:** Countries of Questionnaire Participants With Corresponding Income Groups Indicated Including L, LM, UM, and H Income and Their Respective Number of BT Equipment as Reported by the DIRAC^18^

Country	Income Level	Total BT Equipment (afterloaders)
Zambia	L	2
Bolivia	LM	5
India	LM	317
Indonesia	LM	15
Mongolia	LM	1
Morocco	LM	10
Pakistan	LM	14
Philippines	LM	16
Tunisia	LM	4
Zimbabwe	LM	2
Argentina	UM	44
Brazil	UM	130
Guatemala	UM	7
Malaysia	UM	11
Paraguay	UM	2
South Africa	UM	23
Chile	H	12
Croatia	H	5
The United States	H	771

Abbreviations: BT, brachytherapy; DIRAC, Directory of Radiotherapy Centers; H, high; L, low; LM, lower middle; UM, upper middle.

**FIG 3:**
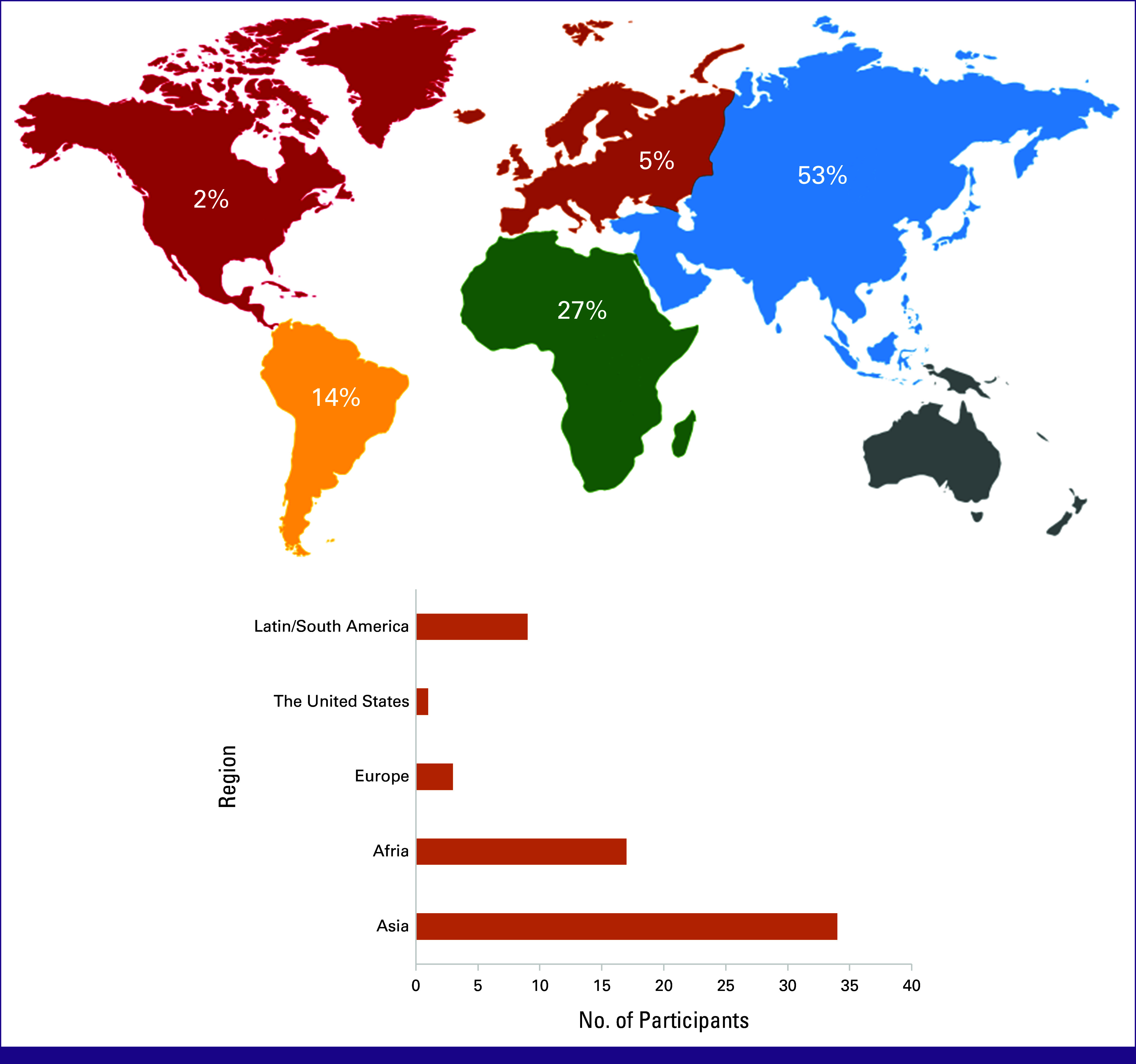
Illustration of the regional participation from Asia Pacific, Africa, Europe, Latin/South America, and North America.

The regional distribution of participants is shown in Figure 3.

**FIG 4:**
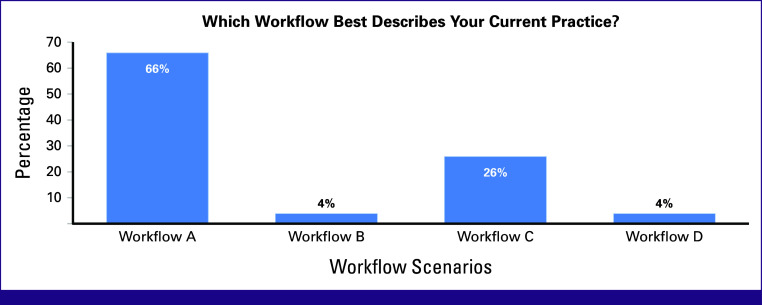
Poll results for the brachytherapy workflow scenarios best describe the current practice of participants of varying institutions.

Most participants (66%) reported scenario A, 26% used scenario C, and 4% used scenarios B and D during routine clinical practice. For most participants, only essential in-room imaging devices such as C-arm imaging and ultrasonography were available, with limited access to additional imaging devices such as CT or MRI (Fig 4).

Other workflow scenarios described by the participants included one similar to scenario C, involving a BT suite. Still, the patient underwent CT or MRI imaging elsewhere within the hospital, following which the patient returned to the suite for planning and treatment. Another workflow described involved sedation and applicator insertion in the operating room, after which the patient was taken for CT and MRI imaging in a different room, the planning was done in the physics room, and treatment was finally done in the BT suite.

**FIG 5:**
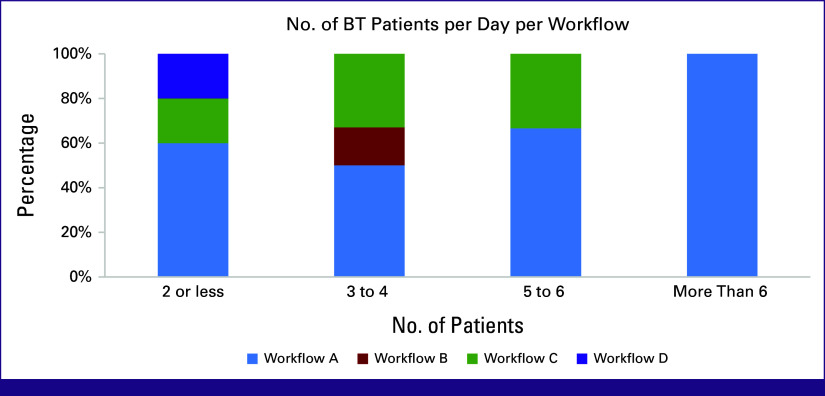
BT workflow scenarios and number of BT patients treated per day. BT, brachytherapy.

Participants reported treating more BT patients with scenario A (47%); those using scenarios A and C treated more than five patients daily. Fifty percent of participants using scenario C reported working up to 12 hours per day compared with only 10% of participants using scenario A. Centers that utilized scenario D reported treating two patients or less (Fig 5).

**FIG 6:**
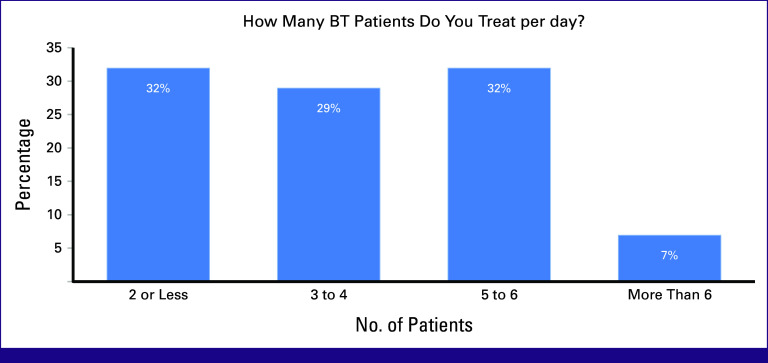
Poll results for the number of BT patients treated daily from a sample of participants from varying institutions. BT, brachytherapy.

Most participants treated between one and six patients daily, with only 7% treating more than six patients per day (Fig 6).

Only 26% of the participants reported that their workflow included overnight stays. These included scenarios where patients were sedated and had applicator insertion performed in the operating theater on day 1, with planning and treatment done on the second day. Another center described overnight inpatient stays for patients who received two treatments.

Most commonly, procedures started between 8 am and 10 am, with no participants reporting work starting after noon. Others reported starting earlier than 8 am in the case of a high patient load or when the source activity was low, resulting in longer treatment times.

**FIG 7:**
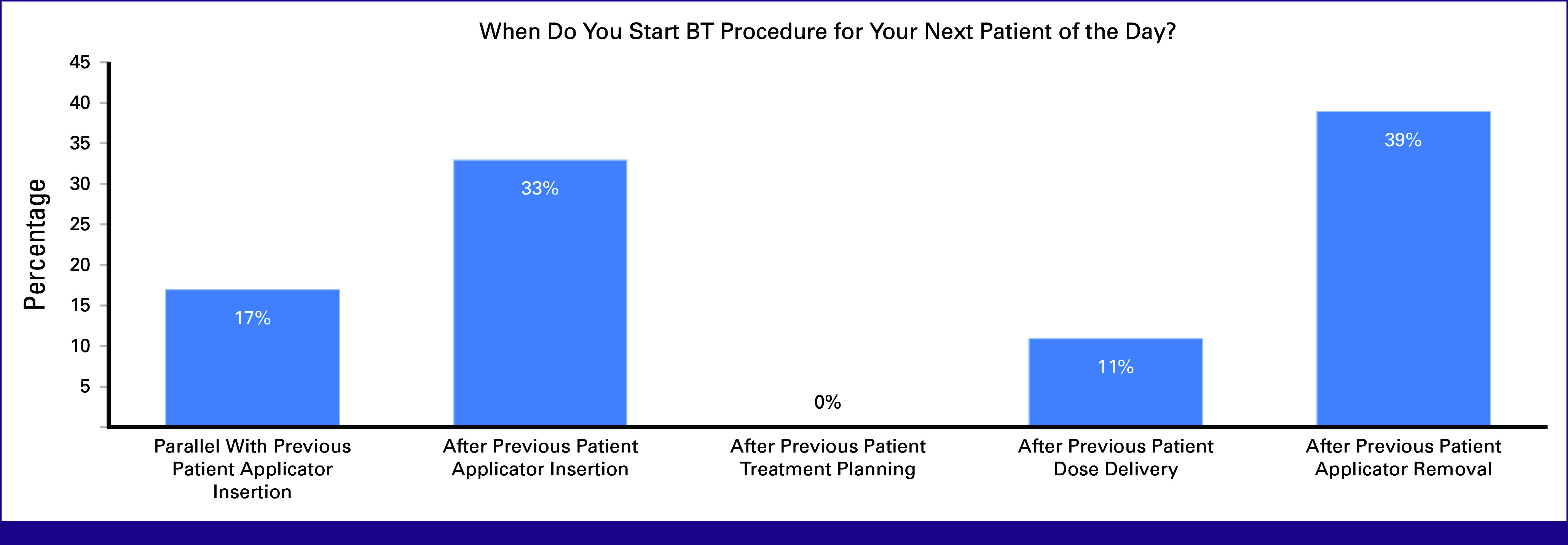
Poll results for the estimated start of the second BT procedure of the day from a sample of participants from varying institutions. BT, brachytherapy.

Thirty-nine percent of the participants started the BT procedure for the next patient after the applicator removal of the previous patient, and 33% started after the previous patient's applicator insertion. Seventeen percent of participants started the workflow for the second patient in parallel with the previous patient's applicator insertion. Participants also reported having separate teams for each patient, with some overlap to allow for parallel patient treatments. Only 11% of patients started the procedure for the next patient after the previous patient's dose delivery. The step of applicator sterilization was also identified as a limitation to treating more patients, as some participants reported requiring sterilization of the same applicators for the next patient. Others reported having multiple sets of applicators sterilized at the end of the day while some alternate triple-channel BT patients with single-channel patients to allow time for applicator sterilization (Fig 7).

It was found that 56% of the units operated between 8 and 12 hours, and 44% worked within 4-8 hours. Each center operated at least 4 hours but not more than 12 hours.

**FIG 8:**
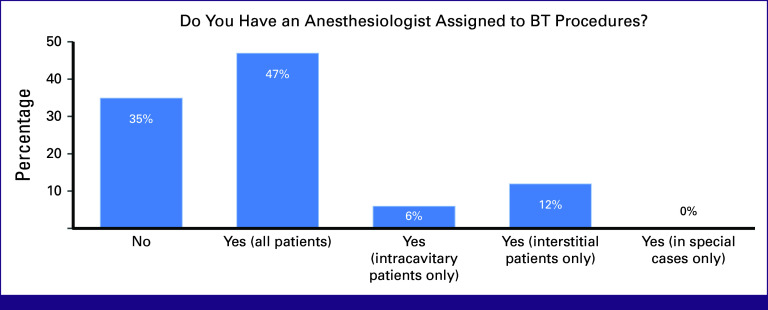
Poll results for the assignment of an anesthesiologist to BT procedures from a sample of participants from varying institutions. BT, brachytherapy.

Anesthesiologists assigned to BT procedures were reported by 47% of the participants while 35% did not have anesthesiologists dedicated to BT. Twelve percent had only anesthesiologists for interstitial BT while 6% had them assigned to intracavitary patients (Fig 8).

## DISCUSSION

IGBT allows physicians to implant and delineate the tumor and organs at risk (OAR) as volumetric structures instead of the conventional point A reference lying a fixed distance from the applicator.^8,19^ Implementation of IGBT also necessitates more individualized implantation techniques using hybrid applicators when indicated. Better outcomes are expected with an improved conformal distribution of dose, modulated to cover the target to an optimal dose while minimizing dose to OAR.^8^ MRI is the gold standard for IGBT because of its excellent soft-tissue contrast and visual accuracy. However, its availability, logistics, and financial implications limit the widespread use of MRI. As a result, alternative imaging techniques such as CT and ultrasound have been explored for IGBT. While CT has been considered helpful for IGBT, using ultrasound for treatment planning has yet to be standardized for this application.^20^ The choice of imaging modality should be based on the individual patient's clinical circumstances and local resources availability. However, IGBT implementation still poses financial, organizational, and technical challenges.^21,22^ Using IGBT requires an enhanced physician skill set, access to cross-sectional imaging, and training of medical physicists and technologists to plan and execute complex procedures. This leads to an increase in the overall cost and time consumption. Kim et al reported 3D IGBT as a cost-effective alternative to 2D BT. However, factors such as time, resources, and availability of expertise should have been considered.^23^ The International Atomic Energy Agency (IAEA) Human Health Report on the transition from 2D to 3D IGBT outlines the indicative cost of 3D BT equipment, including costs of anesthesiology, surgical and other medical equipment, and maintenance.^17^

The design of the operating rooms plays an important role in improving the efficiency of the BT workflow.^22^ Workflow scenarios A and B, practiced by 70% of the participants, presented a scenario in which patients utilized multiple rooms. Combining several steps in different locations to deliver one treatment risks the precision and safety of radiation delivery. Patient transportation can hinder the immobilization of the applicator system.^24^ Interfraction and intrafraction variations due to changes in applicator position during BT imaging, planning, and dose delivery can lead to deviations in the dose delivered.^25,26^ Therefore, BT implant quality (applicator position in relation to target and OAR) needs to be checked and repositioned as needed with available imaging techniques.^27^ Intrahospital transportation of patients for imaging may be due to limited infrastructure. Logistics/administrative issues such as getting time slots for imaging and reimbursement issues may pose a challenge and require good planning. Access to adequate imaging for patients in LMICs remains limited, with 4.3 CT scanners and 1.12 MRI units per million people, with only 0.6 CT scanners and 0.2 MRI units per million in low-income countries, according to the IAEA IMAGINE database.^28^ The installation of CT/MRI scanners within the radiotherapy/BT department requires significant up-front costs and strategic facilities, which may pose a challenge in LMICs.^29,30^

As seen in scenario C, the utilization of a BT suite is typically considered the most efficient workflow scenario with no patient transfer across rooms during the treatment process.^29,31^ The integration of applicator insertion, imaging, treatment planning, and delivery within a single location improves precision by reducing the risk of applicator migration while permitting advanced, IGBT and rapid workflow.^32^ In a study by Anderson et al, time delays during treatment planning and the treatment process were found to have implications for doses to OAR. For bowel, bladder, and rectal doses, the dose to maximally expose 2 cm^3^ of the OAR (D2cc) changed by at least 10% for at least one organ.^33^ However, another study by Muangwong et al^34^ found that compared with in-room brachytherapy, out-of-room brachytherapy did not result in any significant dose change between planning and pretreatment imaging. Nevertheless, it is essential to consider the reduction in patient throughput as a single patient from the examination to dose delivery occupies the BT suite, and this can turn out to be inefficient in high-volume centers. This limits the number of patients who can be treated. In addition, such an arrangement necessitates the availability of anesthesia staff for a longer duration if multiple cases are planned in 1 day. Workflow scenarios A, B, and D in our study allow for workflow overlapping/parallel processing, leading to higher patient throughput. However, the feasibility of simultaneous treatments for multiple patients depends on the availability of BT-allocated staff such as nurses and radiation therapists, the number of available applicator sets including the required cleaning, and sterilization management and BT case management (patient scheduling).^30^ With half of the survey participants delaying the next BT patient scheduling until the previous patient has completed dose delivery and applicator removal, there is potential for enhancing efficiency in these centers through concurrent treatments.

As seen in our results, there is a variation in patient throughput, with most centers treating one to two or five to six patients per day, indicating potential inefficiencies depending on the workflow scenario. Most centers utilizing scenario A exhibited high patient throughput with approximately five to six daily treatments, starting their first procedure between 8 and 10 am. Some centers utilizing scenario C also reported treating more than five patients, which may be attributed to the centers that worked beyond 8 hours per day.

Anesthesia is vital for complex BT procedures such as interstitial BT, often performed for high-risk patients who require both anesthesia and immobilization, allowing for better patient care and pain control.^4,35^ In addition to patient comfort, anesthesia also allows for optimal applicator insertion and vaginal packing that will, in turn, lead to better dose coverage during treatment planning.^4^ In 2015, LMICs were reported to possess only 15% of all anesthesiologists while representing 48% of the global population.^36^ In this study, one third of the participants reported that no anesthesiologist was assigned to BT. The development of accessible training programs in LMICs would be beneficial in increasing the number of trained anesthesiologists in the field of BT.

With multi-stakeholder strategies, the implementation of IGBT could be met through the cooperation of international, nongovernmental, and other organizations while also looking at resource sharing of equipment.^37^ Although effective, IGBT transition is resource intensive, and this may increase the financial burden of institution with limited revenues. An analysis of the transition from 2D to 3D BT in India also highlighted higher costs, the need for more staff, and longer working shifts to accommodate the impact of a transition to more advanced workflows that incorporate image-based BT.^38^

In conclusion, implementing IGBT, especially in LMICs, requires careful planning and consideration of possible factors that impede the efficiency and flow of patients. This study highlights the diversity in clinical practices and resource allocation affecting treatment efficiency and quality. Scenario A (multiple rooms) was the most used as it allowed overlapping of treatment steps leading to more patients being treated per day. The utilization of a BT suite is considered the most efficient; however, performing various steps of the BT procedure in multiple rooms can offer the advantage of parallel preparation of other patients, thereby increasing patient throughput. Therefore, it is essential to develop solutions that address specific needs and limitations of individual institutions to ensure the safe and efficient implementation of IGBT. Government involvement and multi-stakeholder agreements are essential for the shared use of resources such as CT or MRI equipment in BT. In collaboration with the IAEA, WHO, and cancer initiative societies, health authorities should promote BT, especially in LMICs. This advances medical practice and improves women's quality of life globally, thereby contributing to societal well-being.
